# Scorpion Venom Heat-Resistant Synthetic Peptide Alleviates DSS-Induced Colitis via α7nAChR-Mediated Modulation of the JAK2/STAT3 Pathway

**DOI:** 10.3390/antiox14111296

**Published:** 2025-10-28

**Authors:** Kang Cheng, Guangbo He, Xiaxia Li, Yuqian Li, Xiaolin Cui, Xuefei Wu, Jau-Shyong Hong, Jie Zhao, Sheng Li, Yanjie Guo

**Affiliations:** 1 Department of Pathogen Biology and Microecology (Pathogen Biology Laboratory), School of Basic Medical Science, Dalian Medical University, Dalian 116044, China; chengkang@pkuph.edu.cn (K.C.); hegb@dmu.edu.cn (G.H.); lxx_fkx@163.com (X.L.); liyq11@dmu.edu.cn (Y.L.); 2 National-Local Joint Engineering Research Center for Drug-Research and Development (R&D) of Neurodegenerative Diseases, Dalian Medical University, Dalian 116044, China; cuixl01@dmu.edu.cn (X.C.); zhaoj@dmu.edu.cn (J.Z.); 3 Department of Medical Physiology, Dalian Medical University, Dalian 116044, China; wuxf@dmu.edu.cn; 4 Neurobiology Laboratory, National Institute of Environmental Health Sciences, National Institutes of Health, Research Triangle Park, Durham, NC 27709, USA; hong3@niehs.nih.gov; 5 Department of Biochemistry, School of Basic Medical Science, Dalian Medical University, Dalian 116044, China

**Keywords:** IBD, scorpion venom, gut–brain axis, CAIP, α7nAChR, JAK2/STAT3

## Abstract

Background: Inflammatory bowel disease (IBD) is a chronic relapsing inflammatory disorder with limited treatment options. Emerging evidence reveals bidirectional crosstalk between gut and brain through inflammatory signaling, leading us to hypothesize that anti-neuroinflammatory agents may concurrently ameliorate intestinal inflammation. The scorpion venom-derived heat-resistant synthetic peptide (SVHRSP), a bioactive peptide initially identified in scorpion venom and subsequently synthesized by our laboratory, possesses neuroprotective, anti-inflammatory, and antioxidative activities. Its properties make SVHRSP a promising candidate for investigating the therapeutic potential of anti-neuroinflammatory strategies in mitigating intestinal inflammation. Methods: Using a chronic dextran sodium sulfate (DSS)-induced colitis model in wild-type and α7 nicotinic acetylcholine receptor (α7nAChR) knockout mice, along with lipopolysaccharide (LPS)-stimulated RAW264.7 macrophages, we assessed SVHRSP’s effects on inflammation, histopathology, gut permeability, oxidative stress markers, and α7nAChR-Janus kinase 2 (JAK2)/signal transducer and activator of transcription 3 (STAT3) signaling. Results: SVHRSP treatment significantly ameliorated colitis symptoms in wild-type mice by reducing inflammation, repairing histological damage, restoring gut barrier function, and attenuating oxidative stress, with these effects abolished in α7nAChR knockout mice. Mechanistically, SVHRSP activated JAK2/STAT3 signaling through α7nAChR engagement, suppressing proinflammatory cytokine production in macrophages. Conclusion: These results demonstrated that SVHRSP alleviated intestinal inflammation via α7nAChR-dependent JAK2/STAT3 activation. Combined with its known neuroprotective properties, our findings support the repurposing of this neuroactive peptide, SVHRSP, for treating intestinal inflammatory disorders.

## 1. Introduction

Inflammatory bowel disease (IBD), primarily comprising ulcerative colitis (UC) and Crohn’s disease (CD), represents a group of chronic inflammatory disorders characterized by persistent, nonspecific intestinal inflammation [1,2]. The chronic inflammatory process leads to progressive intestinal tissue damage, resulting in debilitating clinical manifestations including abdominal pain, chronic diarrhea, hematochezia, and weight loss. In recent years, epidemiological studies have documented a steady rise in IBD incidence worldwide, posing significant challenges to global healthcare systems [3]. Current therapeutic regimens, including corticosteroids, 5-aminosalicylic acid (5-ASA) derivatives, and immunosuppressants, remain unsatisfactory due to their substantial side effects, limited efficacy in specific patient populations, poor long-term outcomes, and considerable financial burden [4]. These clinical challenges highlight the critical need for developing innovative treatment approaches with improved safety profiles, enhanced therapeutic efficacy, and better cost-effectiveness.

The gastrointestinal and central nervous systems are linked through the sophisticated bidirectional communication network, the gut–brain axis. Inflammatory signals are transmitted between the gut and brain through three principal signaling pathways: the systemic-humoral, cellular immune, and neuronal pathways [5]. Among these, the autonomic nervous system, especially the vagus nerve, is the neural pathway’s core component. The vagus nerve’s afferent fibers relay crucial luminal information (e.g., osmotic changes, mucosal deformation, and microbial metabolites) to the central nervous system. In contrast, its efferent fibers mediate anti-inflammatory responses through the cholinergic anti-inflammatory pathway (CAIP) [6]. This neuroimmunomodulatory circuit involves proinflammatory signal detection by vagal afferents, efferent acetylcholine (ACh) release, and α7 nicotinic acetylcholine receptor (α7 nAChR)-dependent suppression of macrophage-derived cytokines, including interleukin-1 (IL-1), tumor necrosis factor-alpha (TNF-α), and high mobility group box 1 (HMGB1) [7,8,9].

Mounting evidence implicates gut–brain axis dysregulation in IBD pathogenesis. Clinical observations reveal that IBD patients exhibit autonomic dysfunction characterized by vagal hypoactivity and sympathetic predominance [7]. Psychological stressors can exacerbate disease through altered gut motility, enhanced visceral sensitivity, compromised epithelial barrier function, and amplified local inflammation [10]. Notably, vagus nerve stimulation (VNS) therapy demonstrates clinical potential, with studies showing reduced intestinal inflammation in both rodent colitis models and CD patients through its dual anti-inflammatory mechanisms: afferent-mediated hypothalamic–pituitary–adrenal (HPA) axis activation and efferent-mediated CAIP that suppresses TNF-α production [8]. These findings collectively support our hypothesis that pharmacological agents targeting neuroinflammatory pathways (particularly via α7nAChR activation) may confer dual therapeutic benefits for both neural and intestinal inflammatory disorders.

Scorpions have been used in China for thousands of years to treat neurological disorders such as epilepsy and facial paralysis. Our laboratory identified an active peptide sequence consisting of 15 amino acids within the venom of the East Asian pincer scorpion, and we chemically synthesized this specific 15-amino-acid sequence, dubbing it scorpion venom-derived heat-resistant synthetic peptide (SVHRSP) [11,12]. The detailed chemical structure of SVHRSP is the sequence KVLNGPEEEAAAPAE with a molecular weight of 1524.7 Daltons. Previous studies showed that SVHRSP exhibited a favorable safety profile and had a potent anti-inflammatory, antioxidative ability, and neuroprotective effect in the central nervous system [13,14,15]. Notably, SVHRSP has recently received approval from China’s National Medical Products Administration (NMPA) for clinical trials investigating its antiepileptic applications (NMPA Approval No. 2025LP01476, [2025.5.30]), underscoring its established safety profile and drug-like characteristics. Building upon SVHRSP’s demonstrated neuroprotective, anti-inflammatory, and antioxidant properties, and considering the established reciprocity of gut–brain axis signaling, we hypothesized that this neuroactive peptide might exert dual therapeutic effects—ameliorating both central neuroinflammation and peripheral intestinal inflammation through shared α7nAChR-mediated pathways.

This study investigated SVHRSP’s therapeutic potential in dextran sodium sulfate (DSS)-induced chronic colitis, aiming to validate its peripheral anti-inflammatory efficacy and to elucidate its dependence on α7nAChR-Janus kinase 2 (JAK2)/signal transducer and activator of transcription 3 (STAT3) signaling. Our findings offer a novel strategy for repurposing neuroprotective peptide SVHRSP and could expand the therapeutic arsenal for IBD.

## 2. Materials and Methods

### 2.1. Animals

Wild-type (WT) C57BL/6J male mice, aged between 8 and 12 weeks, were sourced from Changsheng Biotechnology (Shenyang, China). α7nAChR-pure knockout (α7 KO) male mice (B6(Cg)-Chrna7tm1.1Ehs/YakelJ, aged between 8 and 12 weeks) were acquired from Jackson Laboratories (Bar Harbor, ME, USA), and the genotype was identified by polymerase chain reaction (PCR) using specific primers (Supplementary Figure S1 and Supplementary Table S1). All mice were fed under standardized conditions (25 ± 2 °C, 50 ± 10% humidity, 12/12 h light/dark cycle). They were provided with unlimited access to food and water throughout the study. All animal experiments were conducted in accordance with the Chinese National Research Council’s Guide for the Care and Use of Laboratory Animals, with the approval of the Institutional Ethics Committee of Dalian Medical University (Ethics Approval No. AEE24086).

### 2.2. Induction and Treatment of Colitis

**Figure 1:**
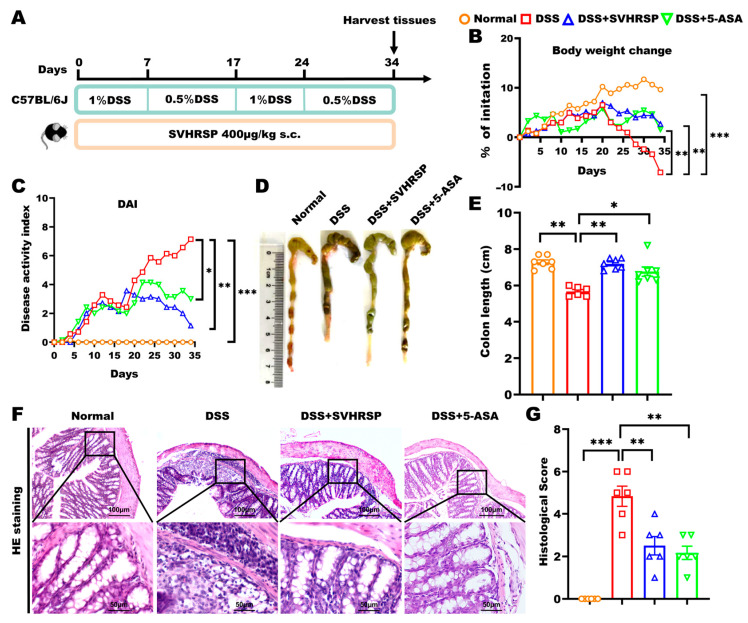
Therapeutic effects of synthetic peptide-scorpion venom heat-resistant synthetic peptide (SVHRSP) in a mouse model of dextran sodium sulfate (DSS)-induced chronic colitis. (**A**) Animal experiment arrangement. (**B**) Changes in body weight (*n* = 6–7). (**C**) Disease Activity Index (DAI) scores (*n* = 6–7). (**D**) Colon pictures and (**E**) length (*n* = 6–7). (**F**) Colon tissue H&E staining and (**G**) scored (*n* = 6). Results were presented as the mean ± standard error of the mean (SEM). * *p* < 0.05, ** *p* < 0.01, and *** *p* < 0.001. 5-ASA, 5-aminosalicylic acid.

The animal experimental design is illustrated in Figure 1A. WT mice were divided into four groups (*n* = 7 each): Normal control, DSS colitis model, DSS+SVHRSP (400 μg/kg/day, s.c.), and DSS+5-ASA (100 mg/kg/day, p.o.). α7 KO mice were divided into three groups: Normal control, DSS model, and DSS+SVHRSP (400 μg/kg/day, s.c.). Chronic colitis was induced through two cycles of 7-day 1% DSS (MP Biomedicals Inc, Santa Ana, CA, USA) followed by 10-day 0.5% DSS in drinking water. The therapeutic dose of SVHRSP (400 μg/kg/day) was selected based on its established efficacy and anti-neuroinflammatory properties demonstrated in our previous studies using Parkinson’s disease models [14]. For administration, SVHRSP was dissolved in sterile normal saline. In contrast, 5-ASA was uniformly suspended in a 0.5% sodium carboxymethyl cellulose (CMC) solution due to its poor aqueous solubility. Treatments were administered daily throughout the study, with SVHRSP delivered subcutaneously and 5-ASA by oral gavage. DSS solution and therapeutic agents were prepared fresh daily to ensure efficacy.

### 2.3. Disease Activity Index (DAI)

The DAI scoring system evaluates the severity of colitis by assessing three parameters: the percentage of body weight loss, the characteristics of the feces, and the blood in the feces. The total DAI score was the aggregate of these three individual scores, with the specific scoring criteria provided in Supplementary Table S2 [16].

### 2.4. Histological Analysis

Mice were euthanized, and a 1 cm section of their terminal colon was fixed in 4% (*w*/*v*) paraformaldehyde (PFA, Biosharp, Shanghai, China), then processed into paraffin blocks and sectioned for staining with hematoxylin and eosin (H&E). The histological evaluation of colonic H&E staining was as follows: (a) inflammatory cell infiltration was scored as follows: none = 0; slight = 1; moderate = 2; severe = 3, and (b) tissue damage was scored based on the depth of injury: none = 0; mucosal layer = 1; submucosal layer = 2; muscularis propria = 3; and transmural = 4, with the final score being the sum of the scores from both categories (a) and (b) [17].

### 2.5. Fluorescein Isothiocyanate (FITC)-Dextran Osmotic Test

Mice were fasted for 12 h and then given a gavage of 400 mg/kg FITC-dextran (molecular weight: 3–5 kDa, Aladdin, Shanghai, China). After 4 h, serum from mice was collected, and the fluorescence intensity was detected using a multifunctional enzyme labeling instrument (excitation light 485 nm; emission light 528 nm).

### 2.6. Reverse Transcription Quantitative Polymerase Chain Reaction (RT-qPCR)

Colonic tissue and cellular RNA were extracted utilizing a TRIzol reagent kit (Vazyme Biotech, Nanjing, China). cDNA synthesis and qPCR analysis were performed using a cDNA synthesis kit and SYBR Green reagent (Vazyme Biotech, Nanjing, China). Gene expression levels were standardized against glyceraldehyde-3-phosphate dehydrogenase (GAPDH) and quantified using the 2^−ΔΔCt^ method. The reagents, primers, and temperature cycles for RT-qPCR are shown in Supplementary Tables S3–S5.

### 2.7. Western Blot

Protein extracts from mouse colon tissues and RAW264.7 cells were prepared using RIPA lysis buffer (Solarbio, Beijing, China). Following separation and transfer to nitrocellulose membranes, samples were probed with the following primary antibodies: GAPDH at a 1:1000 dilution (Servicebio, Wuhan, China), zonula occludens-1 (ZO-1) at a 1:500 dilution, Occludin at a 1:1000 dilution, α7 nicotinic acetylcholine receptor at a 1:1000 dilution, JAK2 at a 1:1000 dilution, P-JAK2 at a 1:500 dilution, STAT3 at a 1:1000 dilution, and P-STAT3 at a 1:1000 dilution (Abcam, Cambridge, UK), nicotinamide adenine dinucleotide phosphate oxidase 2 (NOX2) at a 1:1000 dilution (Proteintech, Wuhan, China), and incubation with horseradish peroxidase (HRP)-conjugated secondary antibody (1:10,000 dilution, Abbkine, Wuhan, China). Images were obtained with a BioRad gel imaging system and analyzed with ImageJ (1.8.0) software.

### 2.8. Alcian Blue Staining

Alcian Blue staining was performed to visualize acidic mucins. Colon tissue sections (5 µm) were deparaffinized, rehydrated through a graded ethanol series, and stained with Alcian Blue solution (pH 2.5) for 30 min at room temperature. After rinsing with distilled water, the sections were counterstained with nuclear fast red for 5 min. Finally, the sections were dehydrated, cleared in xylene, and coverslipped. Mucin staining intensity was assessed under a light microscope and quantified using Image J (1.8.0) software.

### 2.9. Immunohistochemistry

Colonic tissue sections of 5 µm thickness were processed to remove paraffin, underwent antigen retrieval, and were then blocked to prevent nonspecific binding. They were then incubated with the specified primary antibodies overnight at 4 °C: α7 nicotinic acetylcholine receptor at a 1:500 dilution (Abcam, Cambridge, UK), F4/80 at a 1:250 dilution (CST, Danvers, MA, USA), mucin 2 (MUC2) at a 1:200 dilution, ZO-1 at a 1:100 dilution, Occludin at a 1:200 dilution, and 8-hydroxy-2′-deoxyguanosine (8-OHdG) at a 1:200 dilution (Abcam, Cambridge, UK). The next day, the sections were treated with biotinylated secondary antibodies (ZSGB, Beijing, China) for 1 h at room temperature. The sections were incubated with streptavidin conjugated to horseradish peroxidase (ZSGB, Beijing, China) for another hour at room temperature. The sections were visualized using 3, 3′-diaminobenzidine (DAB) staining and counterstained with hematoxylin. The sections were scanned and analyzed under Pannoramic MIDI (3DHistech, Budapest, Hungary). The average optical density (AOD) was measured using Image J (1.8.0) software.

### 2.10. Flow Cytometry

Freshly harvested mouse colon tissues were immediately rinsed with precooled phosphate-buffered saline (PBS) at 4 °C, then cut into pieces, added with EDTA (5 mM) solution containing 5% fetal bovine serum (FBS), and digested at 37 °C, 300 rpm shaker for 40 min. The supernatant was filtered using a sterile cloth (100-mesh). The supernatant was recovered by adding the digestion solution containing collagenase IV, DNase I, and Dispase I to the sediment (Worthington, Lakewood, NJ, USA) to the residue for 25 min at 37 °C with shaking at 300 rpm. The supernatant was recovered using a sterile filter cloth (200-mesh) to obtain a single-cell suspension of colonic tissue. The isolated single cells were resuspended and then incubated with the appropriate antibodies, anti-F4/80 PERCP and anti-CD11b FITC (Biolegend, San Diego, CA, USA) at 4 °C for 30 min in the dark. Flow cytometry detected and recorded the fluorescence signals (NovoCyte, San Diego, CA, USA). Subsequently, the data were analyzed using Novoexpress and visualization. The detailed gating strategy and unstained control for flow cytometry analysis of macrophages are provided in Supplementary Figures S2 and S3.

### 2.11. Biochemical Assays for Oxidative Stress

Colon tissue samples were homogenized in ice-cold saline and centrifuged at 12,000× *g* for 15 min at 4 °C to collect the supernatant. Levels of oxidative stress markers were evaluated using commercially available assay kits (Nanjing Jiancheng Bioengineering Institute, Nanjing, China) following the instructions provided by the manufacturer. Catalase (CAT) activity was assessed by monitoring the decomposition of hydrogen peroxide (H_2_O_2_). The activity of superoxide dismutase (SOD) was analyzed via the water-soluble tetrazolium salt-1 (WST-1) method, which measures the inhibition of superoxide anion (O_2_•^−^)-driven formazan generation. The concentration of malondialdehyde (MDA), an indicator of lipid peroxidation, was quantified using the thiobarbituric acid (TBA) method. Myeloperoxidase (MPO) activity was assayed by detecting the peroxidase-catalyzed oxidation of a chromogenic substrate in the presence of H_2_O_2_.

### 2.12. Peritoneal Macrophage Isolation and Dihydroethidium (DHE) Staining

Peritoneal macrophages were isolated from WT mice euthanized by cervical dislocation. The peritoneal cavity was lavaged with 10 mL of sterile PBS after gentle abdominal massage for 2 min. The collected lavage fluid was filtered through a 70-μm strainer and centrifuged at 400× *g* for 5 min. The resulting cell pellet was resuspended in culture medium and allowed to adhere for 2 h at 37 °C under 5% CO_2_. After removing non-adherent cells by washing with warm PBS, the primary macrophages were divided into three groups: control (untreated), DSS (2.5% DSS for 30 min), and SVHRSP (30 μM SVHRSP pretreatment for 30 min followed by DSS exposure). The SVHRSP pretreatment concentration of 30 μM was selected based on its established efficacy in our prior research, which demonstrated significant suppression of microglial activation at this concentration, and considering that microglia represent the resident macrophage population of the central nervous system [14]. Following treatment, cells were incubated with Dihydroethidium (DHE) at 37 °C for 30 min in the dark, and fluorescence was visualized using fluorescence microscopy.

### 2.13. Enzyme-Linked Immunosorbent Assay (ELISA)

The concentration of acetylcholine (ACh) in colon tissue was measured from the supernatant of tissue homogenates. Briefly, 30 mg of colon tissue was precisely weighed, and 270 µL of ice-cold PBS (pH 7.2–7.4) was added, achieving a fixed weight-to-volume ratio of 1:9. The tissue was homogenized on ice using a tissue grinder. The resulting homogenate was centrifuged at 2000 rpm for 20 min, and the supernatant was collected for ACh analysis. This standardized protocol, using the same amount of starting tissue for all samples, ensured that the measured ACh concentration accurately reflected the level in the original tissue, enabling valid comparisons between experimental groups. ACh levels in the prepared colonic tissue supernatants were quantified using an ELISA kit from Meimian, Jiangsu, China, and the levels of interleukin-6 (IL-6), TNF-α, and interleukin-1 beta (IL-1β) in serum samples were quantified using ELISA kits from Elabscience, Wuhan, China, following the instructions provided by the manufacturer.

### 2.14. Cell Culture and Processing

The murine macrophage-like cell line RAW264.7 (purchased from the Chinese Academy of Medical Sciences cell center) was used in this study to investigate the impact of SVHRSP on α7nAChR expression and its anti-inflammatory effects in a controlled in vitro setting. This cell line, originally derived from a tumor induced by Abelson murine leukemia virus (A-MuLV) in a BALB/c mouse, is widely used as a model for studying macrophage biology and inflammatory responses [18]. The RAW264.7 cells were grown in DMEM (Gibco, Waltham, USA), enriched with 10% FBS and 1% penicillin-streptomycin, and cultured at 37 °C with 5% CO_2_. The study involved four groups: the control group, the lipopolysaccharide (LPS) group (100 ng/mL, for 24 h) (Sigma, Louis, MO, USA), the SVHRSP group (pre-treated with 30 μM SVHRSP for 30 min, before exposure to LPS), and the positive control nicotine group (100 nM nicotine for 30 min, and subsequently exposed to LPS) (Aladdin, Shanghai, China).

### 2.15. Molecular Docking

The molecular docking of SVHRSP with α7nAChR was performed to predict their potential interaction. The three-dimensional structure of α7nAChR (UniProt ID: P22770) was obtained from the AlphaFold database. The structure of SVHRSP was generated and energy-minimized using Open Babel at pH 7.4 [19]. Docking simulations were carried out with AutoDock Vina (version 1.2.0), with the receptor and ligand prepared using AutoDock Tools [20,21]. A grid box encompassing the potential binding site was defined using AutoGrid. The docking pose with the most favorable binding energy was selected for subsequent interaction analysis. Molecular visualization and analysis of interactions (hydrogen bonds, ionic, and hydrophobic) were conducted using PyMOL (version 2.5).

### 2.16. Statistical Analysis

Data were presented as the mean ± standard error of the mean (SEM). Body weight changes and DAI scores were evaluated using two-way ANOVA with Sidak post hoc analysis for statistical analysis, while other parameters were assessed by one-way ANOVA followed by Tukey’s multiple comparisons test, utilizing GraphPad Prism version 9.0 software (San Diego, CA, Canada). Statistical significance was defined as *p* < 0.05.

## 3. Results

### 3.1. SVHRSP Ameliorated DSS-Induced Colitis in Mice

IBD is characterized by recurrent episodes of chronic nonspecific inflammation of the intestine. To simulate this condition, we established a chronic colitis model by administering alternating cycles of 1% and 0.5% DSS in drinking water over 34 days. During this time, SVHRSP was administered subcutaneously daily. A positive control group treated with 5-aminosalicylic acid (5-ASA) was also included for comparison (Figure 1A). Both SVHRSP and 5-ASA treatments demonstrated significant therapeutic effects, as evidenced by improved disease parameters: attenuation of body weight loss (*p* < 0.01 vs. DSS group), preservation of colon length (*p* < 0.01), and reduction in DAI scores (*p* < 0.01) (Figure 1B–E). Histological analysis showed that SVHRSP and 5-ASA treatments significantly reduced inflammatory cell infiltration (*p* < 0.01), decreased tissue damage scores (*p* < 0.05), and restored crypt architecture (Figure 1F,G). These findings demonstrated that SVHRSP effectively mitigated both the symptoms and histopathological manifestations of chronic colitis in mice, with its overall efficacy surpassing that of 5-ASA.

### 3.2. SVHRSP Preserved the Integrity of the Intestinal Barrier in Colitis Induced by DSS

**Figure 2:**
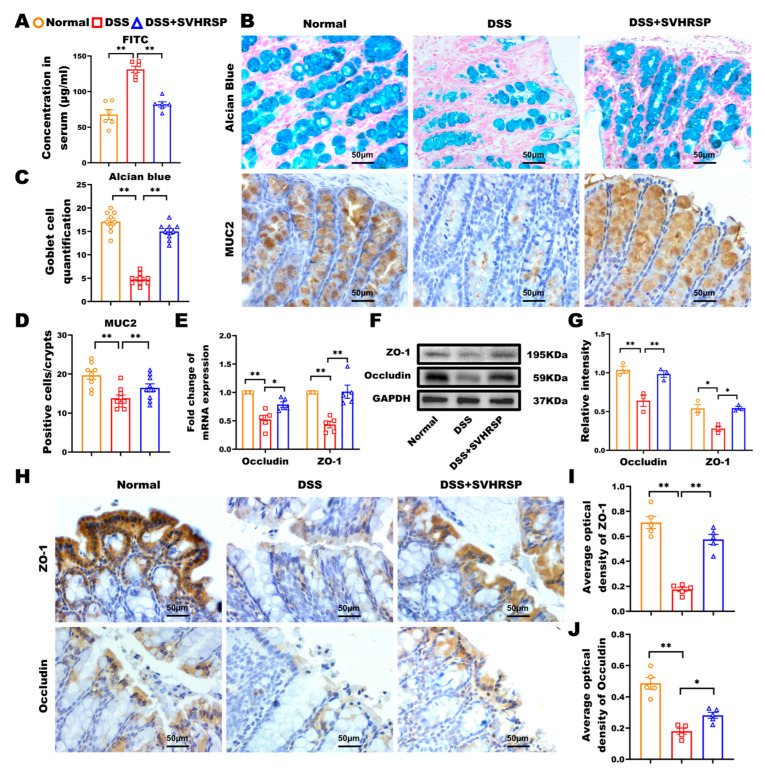
Synthetic peptide-scorpion venom heat-resistant synthetic peptide (SVHRSP) protected the integrity of the intestinal barrier in mice with colitis. (**A**) Fluorescein isothiocyanate (FITC)-dextran (4 kDa) detection of intestinal permeability (*n* = 6). (**B**) Alcian blue staining to observe mucin (blue) secreted by goblet cells in the colon tissues, with the pink color serving as the nuclear counterstain. Immunohistochemistry staining to detect the expression of mucin 2 (MUC2) (brown) in colon tissues (*n* = 3). (**C**) The number of Alcian blue-positive cells was counted by randomly selecting nine crypts in each section, three in each group. (**D**) MUC2-positive cells per crypt (*n* = 9). (**E**) Reverse transcription quantitative polymerase chain reaction (RT-qPCR) analysis of Occludin and zonula occludens-1 (ZO-1) (*n* = 5). (**F**) Western blot analysis of Occludin and ZO-1 and (**G**) quantitative analysis in colon tissues (*n* = 3). (**H**) Immunohistochemistry staining analysis of Occludin and ZO-1 (brown) in colon tissues and (**I**,**J**) quantitative analysis (*n* = 3). Results were expressed as mean ± standard error of the mean (SEM). * *p* < 0.05, ** *p* < 0.01.

The integrity of the intestinal epithelium’s mechanical barrier is closely linked to the progression of colitis [22]. We comprehensively evaluated barrier function through multiple complementary approaches. FITC-dextran is a fluorescently labeled polysaccharide that can pass the intestinal barrier into the bloodstream. Thus, the serum FITC-dextran fluorescence intensity could represent the intestinal barrier function. In this study, using FITC-dextran translocation assays, we observed significantly reduced serum fluorescence intensity in SVHRSP-treated mice compared to DSS controls (*p* < 0.01, Figure 2A), demonstrating improved barrier function. The glycoprotein MUC2 secreted by the goblet cells and the tight junction proteins between intestinal epithelial cells are the primary regulators of the mucosal barrier function [23]. To evaluate further, we performed Alcian blue staining and MUC2 immunohistochemistry to identify the number of goblet cells and mucins. Our results showed that SVHRSP prevented the DSS-induced depletion of Alcian blue-positive cells (*p* < 0.05 vs. DSS, Figure 2B–D) and SVHRSP maintained MUC2 expression levels (*p* < 0.01, Figure 2E). Additionally, the SVHRSP intervention attenuated the DSS-induced reduction in tight junction proteins Occludin and ZO-1 (Figure 2F–J). These findings collectively demonstrated that SVHRSP protected against DSS-induced barrier dysfunction through reducing paracellular permeability, maintaining mucin production, and preserving tight junction architecture.

### 3.3. SVHRSP Attenuated Macrophage-Mediated Inflammation in DSS-Induced Colitis

**Figure 3:**
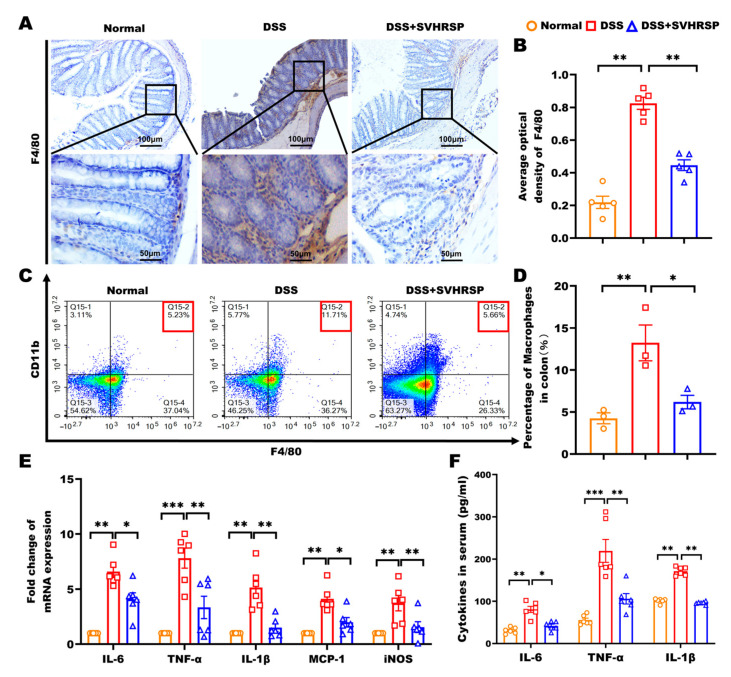
Synthetic peptide-scorpion venom heat-resistant synthetic peptide (SVHRSP) reduced macrophage accumulation and the secretion of inflammatory mediators in colitis mice. (**A**,**B**) Immunohistochemistry analyses of F4/80^+^ cells (brown) in the colonic tissues and quantitative analysis (*n* = 4). (**C**,**D**) Flow cytometry analyses of F4/80^+^CD11b^+^ labeled macrophages in colonic tissues (*n* = 3). (**E**) Reverse transcription quantitative polymerase chain reaction (RT-qPCR) was used to assess the mRNA levels of interleukin-6 (IL-6), tumor necrosis factor-alpha (TNF-α), interleukin-1 beta (IL-1β), monocyte chemoattractant protein-1 (MCP-1), and inducible nitric oxide synthase (iNOS) in colon tissues (*n* = 6). (**F**) Enzyme-linked immunosorbent assay (ELISA) was employed to measure the serum levels of IL-6, TNF-α, and IL-1β in the mice (*n* = 6). Results were expressed as mean ± standard error of the mean (SEM). * *p* < 0.05, ** *p* < 0.01 and *** *p* < 0.001.

Given the pivotal role of macrophages in colitis pathogenesis through excessive proinflammatory cytokine production [24,25]. We systematically evaluated the effects of SVHRSP on macrophage infiltration and activation. Immunohistochemical analysis revealed that SVHRSP treatment significantly reduced F4/80^+^ macrophage accumulation in colonic tissues compared to DSS controls (*p* < 0.01, Figure 3A,B). Flow cytometry demonstrated a marked decrease in CD11b^+^ F4/80^+^ macrophage populations following SVHRSP intervention (vs DSS, *p* < 0.05, Figure 3C,D). In line with the reduction in macrophage infiltration, both the mRNA expression of proinflammatory cytokines in colonic tissues and their serum levels were significantly reduced in the SVHRSP group compared to the DSS group (Figure 3E,F). These results demonstrated that SVHRSP exerted potent anti-inflammatory effects in colitis through limiting macrophage recruitment to inflamed tissue, reducing activation of tissue-resident macrophages, and suppressing downstream proinflammatory cytokine production.

### 3.4. SVHRSP Modulated Oxidative Stress and Protected Against DSS-Induced Colitis

**Figure 4:**
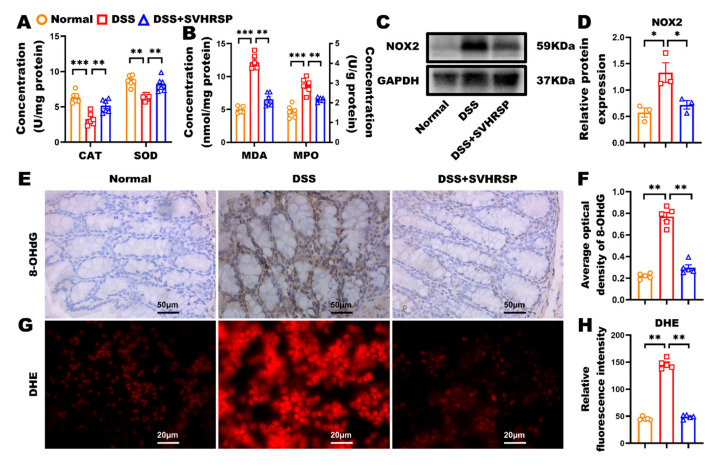
Synthetic peptide-scorpion venom heat-resistant synthetic peptide (SVHRSP) modulated oxidative stress and reactive oxygen species (ROS) production in dextran sodium sulfate (DSS)-induced colitis. (**A**,**B**) The colonic concentrations of catalase (CAT), superoxide dismutase (SOD), malondialdehyde (MDA), and myeloperoxidase (MPO) (*n* = 6). (**C**) Western blot analysis of nicotinamide adenine dinucleotide phosphate oxidase 2 (NOX2) and (**D**) quantitative analysis in colon tissues (*n* = 3). (**E**) Immunohistochemistry staining analysis of 8-hydroxy-2′-deoxyguanosine (8-OHdG) (brown) in colon tissues and (**F**) quantitative analysis (*n* = 5). (**G**) SVHRSP pre-treatment of wild-type (WT) peritoneal macrophages to analyze ROS production and (**H**) quantitative analysis of the dihydroethidium (DHE)-positive area (*n* = 5). Results were expressed as mean ± standard error of the mean (SEM). * *p* < 0.05, ** *p* < 0.01 and *** *p* < 0.001.

Excessive oxidative stress is one of the key factors contributing to the development of IBD, playing a pivotal role in exacerbating intestinal inflammation and tissue damage. To investigate the potential protective effect of SVHRSP on oxidative stress in IBD, we assessed various oxidative stress markers in the colon, including antioxidant enzyme activity, lipid peroxidation, and oxidative DNA damage. Our results demonstrated that SVHRSP treatment ameliorated DSS-induced oxidative imbalances. Specifically, SVHRSP preserved the levels of CAT and SOD, key antioxidant enzymes, while significantly reducing the levels of MDA and MPO, both of which are markers of oxidative damage (Figure 4A,B). Further analysis of NADPH oxidase subunit NOX2 revealed that SVHRSP treatment markedly reduced its elevated levels, indicating a reduction in reactive oxygen species (ROS) production (Figure 4C,D). Moreover, oxidative DNA damage, as indicated by elevated 8-OHdG levels, was significantly increased in DSS-treated mice. However, SVHRSP treatment effectively reduced the 8-OHdG-positive area in colonic tissues, suggesting its protective effect on DNA integrity (Figure 4E,F). In addition, peritoneal macrophages exposed to DSS showed elevated superoxide production, which was quantified by measuring the fluorescence intensity of the DHE oxidation product. SVHRSP treatment significantly reduced DSS-induced ROS production in these macrophages (Figure 4G,H). These data suggest that SVHRSP may exert its protective effect by modulating systemic and local oxidative stress levels, thus attenuating the inflammatory response associated with IBD. These findings highlight the potential of SVHRSP as a therapeutic agent for mitigating oxidative stress and inflammation in IBD.

### 3.5. SVHRSP Attenuated Intestinal Inflammation Through α7nAChR-Mediated Cholinergic Anti-Inflammatory Pathway

**Figure 5:**
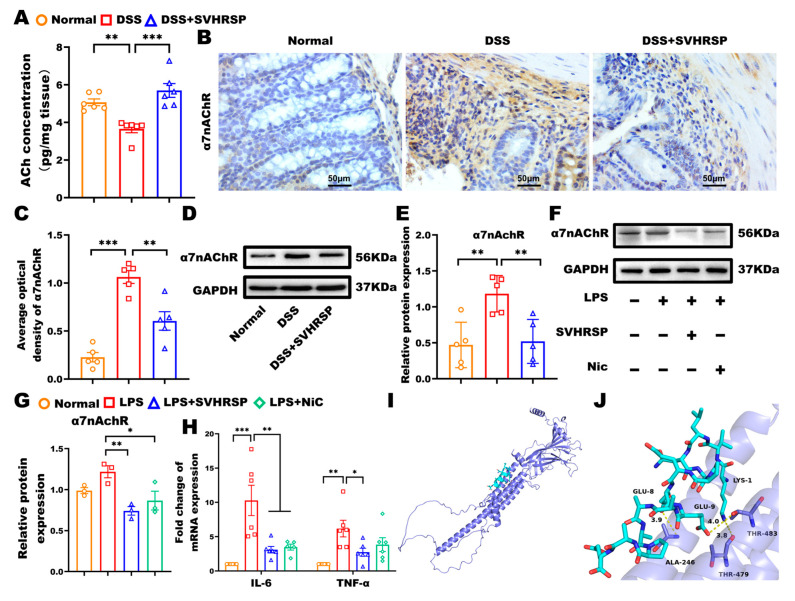
Synthetic peptide-scorpion venom heat-resistant synthetic peptide (SVHRSP) inhibited dextran sodium sulfate (DSS)-induced chronic colitis in mice via a cholinergic anti-inflammation pathway. (**A**) Expression of acetylcholine (Ach) in colonic tissues by enzyme-linked immunosorbent assay (ELISA) (*n* = 6). (**B**) Immunohistochemistry analysis of α7 nicotinic acetylcholine receptor (α7nAChR) (brown) in colon tissues and (**C**) quantitative analysis (*n* = 5). (**D**) Western blot analysis of α7nAChR and (**E**) quantitative analysis in colon tissues (*n* = 5). The original blots are shown in Supplementary Figure S3. (**F**) Western-blot analysis of α7nAChR in RAW264.7 cells and (**G**) quantitative analysis (*n* = 3). The original blots are shown in Supplementary Figure S4. (**H**) Reverse transcription quantitative polymerase chain reaction (RT-qPCR) was used to measure mRNA levels of interleukin-6 (IL-6) and tumor necrosis factor-α (TNF-α) in RAW264.7 cells (*n* = 6). (**I**) Overall docking model of SVHRSP with α7nAChR. (**J**) Enlarged view showing hydrogen-bond interactions with residues THR483, THR479, and ALA246. α7nAChR is depicted as a slate cartoon, SVHRSP as cyan sticks, binding site residues as magenta sticks, and hydrogen bonds as yellow dashed lines. Predicted binding energy is –5.4 kcal/mol. Results were expressed as mean ± standard error of the mean (SEM). * *p* < 0.05, ** *p* < 0.01 and *** *p* < 0.001.

The CAIP represents a critical neural mechanism for regulating systemic inflammation, with α7nAChR-expressing macrophages serving as key cellular mediators [26,27]. To investigate SVHRSP’s mechanism of action, we performed comprehensive analyses of the CAIP components. DSS-induced colitis was associated with significant depletion of colonic ACh levels (vs control, *p* < 0.01), concurrent α7nAChR upregulation (*p* < 0.01), and suggested compensatory receptor overexpression. SVHRSP treatment normalized these perturbations, increasing ACh (*p* < 0.05) while reducing α7nAChR expression (Figure 5A–E). To further determine that α7nAChR was the target in SVHRSP treatment, we analyzed the expression of α7nAChR in macrophage RAW264.7 cells. The results showed that SVHRSP mirrored the effects of nicotine (positive control), reducing α7nAChR expression (*p* < 0.01 vs. LPS). Both treatments significantly suppressed LPS-induced inflammatory cytokines IL-6 and TNF-α (*p* < 0.05) (Figure 5F–H).

To obtain direct computational evidence for the interaction between SVHRSP and α7nAChR, we performed molecular docking analysis. The results demonstrated that SVHRSP could bind stably to α7nAChR with a favorable binding energy of −5.4 kcal/mol. As shown in Figure 5I,J, specific hydrogen bonds were formed between SVHRSP and the key residues THR483, THR479, and ALA246 of α7nAChR. This predicted binding mode provides a structural basis for the binding and potential activation of α7nAChR by SVHRSP. This potential interaction functionally correlates with the α7nAChR-dependent anti-inflammatory effects demonstrated in subsequent experiments using α7nAChR-knockout mice.

### 3.6. Knockout of α7nAChR Abolished the Therapeutic Effects of SVHRSP in Colitis

**Figure 6:**
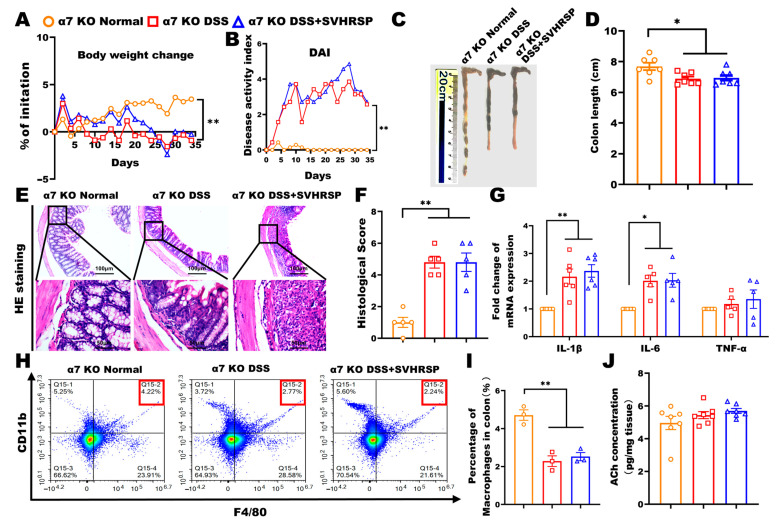
Knockout of α7 nicotinic acetylcholine receptor (α7nAChR) attenuated the protective effect of synthetic peptide-scorpion venom heat-resistant synthetic peptide (SVHRSP). (**A**) The Body weight changes, (**B**) disease activity index (DAI) scores, (**C**,**D**) colon length (*n* = 7). (**E**,**F**) Colon tissue H&E staining and scored (*n* = 4). (**G**) Reverse transcription quantitative polymerase chain reaction (RT-qPCR) analysis of the mRNA expression levels of interleukin-1 beta (IL-1β), interleukin-6 (IL-6) and tumor necrosis factor-alpha (TNF-α) in colon tissues (*n* = 6). (**H**,**I**) Flow cytometry analysis of F4/80^+^CD11b^+^ labeled macrophages in colon tissues (*n* = 3). (**J**) Level of Acetylcholine (Ach) in colon tissues by enzyme-linked immunosorbent assay (ELISA) (*n* = 7). The data were expressed as the mean ± standard error of the mean (SEM). * *p* < 0.05, ** *p* < 0.01.

To establish the critical dependence of SVHRSP’s protective effects on α7nAChR signaling, we conducted parallel experiments in α7nAChR knockout (KO) mice. To verify the essential role of α7nAChR in treating colitis with SVHRSP, we performed DSS-induced chronic colitis in α7nAChR KO mice. The results were in contrast to WT mice, and the SVHRSP treatment did not ameliorate colitis mice’s symptoms and colonic histopathology (Figure 6A–F). The SVHRSP treatment failed to inhibit macrophage accumulation and reduce inflammatory cytokine levels in the colon tissues of DSS-exposed mice (Figure 6G–I). Moreover, the concentration of ACh was not significantly different among the three groups of mice (Figure 6J). These data demonstrated that the deletion of the α7nAChR gene abolished the treatment effect of SVHRSP on DSS-induced colitis, indicating that α7nAChR is a target of SVHRSP.

### 3.7. SVHRSP Suppressed Inflammation via α7nAChR Mediated JAK2/STAT3 Pathway

**Figure 7:**
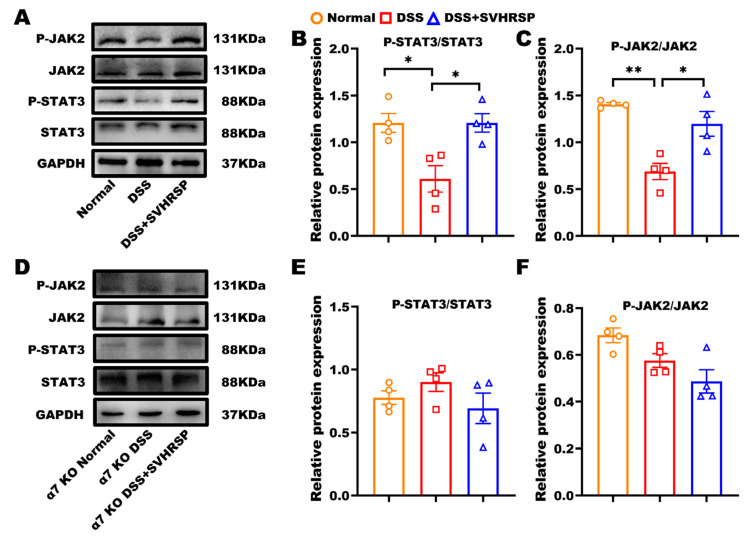
Synthetic peptide-scorpion venom heat-resistant synthetic peptide (SVHRSP) suppressed inflammation by activating the Janus kinase 2 (JAK2)/signal transducer and activator of transcription 3 (STAT3) signaling pathway. Western-blot analysis of JAK2/STAT3 and quantified in wild-type (WT) mice (**A**–**C**) and in α7 nicotinic acetylcholine receptor (α7nAChR) KO mice (**D**–**F**) (*n* = 4). Results were expressed as mean ± standard error of the mean (SEM). * *p* < 0.05, ** *p* < 0.01.

To study the molecular pathways involved in SVHRSP treatment, we examined the JAK2/STAT3 signaling pathway, which is implicated in the cholinergic anti-inflammatory pathway [28]. The results revealed that the phosphorylated JAK2 and STAT3 were reduced in the DSS group, whereas SVHRSP treatment activated JAK2 and STAT3 in WT mice (Figure 7A–C). In contrast, the activation of JKA2 and STAT3 phosphorylation by SVHRSP treatment was blocked in α7 KO mice (Figure 7D–F). These data suggested that SVHRSP exerted anti-inflammatory effects by activating the α7nAChR-mediated JAK2/STAT3 pathway.

## 4. Discussion

The gut–brain axis exhibits bidirectional therapeutic reciprocity, wherein pharmacological agents targeting one system exert beneficial effects on the other. This phenomenon is particularly evident in inflammatory conditions, where neuroactive compounds, such as antidepressants, antipsychotics, and antiepileptics, exhibit significant anti-inflammatory effects in the gastrointestinal (GI) tract. For instance, fluoxetine, a selective serotonin reuptake inhibitor (SSRI), alleviates intestinal inflammation by remodeling intestinal cells and macrophages, suggesting its therapeutic potential for IBD [29]. Similarly, the antidepressant clomipramine reduces ulcerative colitis inflammation in rat models [30]. Levetiracetam is an antiepileptic drug that mitigates colitis in mice by regulating inflammatory and oxidative responses, further underscoring the role of neuroactive drugs in GI inflammation [31]. Conversely, GI-targeting therapies demonstrate significant neuroprotective potential, exemplifying the bidirectional nature of gut–brain axis interventions. Clinically established anti-TNF-α agents like infliximab, while primarily used for IBD, have shown efficacy in improving traumatic brain injury (TBI) [32]. Similarly, probiotic therapies targeting gut microbiota composition have emerged as promising adjunct treatments for depression, anxiety disorders, and Parkinson’s disease [33].

SVHRSP was derived from an active peptide found in Buthus martensii Karsch (Bmk), an animal-based traditional Chinese medicine (TCM) commonly used to treat neurological disorders. Previous studies have shown that SVHRSP exhibited multi-target neuroprotection by inhibiting NADPH oxidase/NLRP3 inflammasome and modulating microglial Nav1.6 channels to protect dopaminergic neurons in Parkinson’s disease [13,14,34], reducing oxidative stress and neuroinflammation in cognitive decline [15], and regulating NMDA receptors to exert antiepileptic effects [35]. This study investigated the novel neuroactive peptide SVHRSP’s impact on DSS-induced colitis in mice.

In our experiments, to simulate the long-term chronic course of disease in IBD patients, a model of chronic colitis was established by alternately giving mice 1% and 0.5% DSS solution [36]. Our findings indicated that SVHRSP treatment improved the colitis symptoms and reduced colonic histopathological scores. In addition, SVHRSP markedly attenuated oxidative stress injury, as evidenced by decreased lipid peroxidation and enhanced antioxidant enzyme activities, thereby alleviating colonic inflammation and tissue damage. Alterations in intestinal permeability are closely associated with the pathogenesis of UC [37,38]. Losing tight junction protein and mucin production increases the gut’s permeability, forming a leaky gut. Translocation of microbes and their toxic metabolites beyond the gut triggers immune cell activation and produces inflammatory cytokines such as IL-6, IL-1β, and TNF-α [24,39]. The SVHRSP treatment increased tight junction protein expression, protected the goblet cells and the secretion of MUC2, and reduced intestinal permeability, suggesting that SVHRSP could potentially inhibit colitis by restoring intestinal integrity.

Intestinal macrophages are distributed throughout the gastrointestinal tract and are the largest macrophage population in the body. In inflammatory bowel disease, these macrophages undergo pathological activation, producing substantial proinflammatory factors, which are thought to underlie the chronic inflammation in IBD [40]. Reducing macrophage activation and their production of inflammatory mediators helps mitigate intestinal damage in IBD. In our study, we found that SVHRSP treatment decreased the number of macrophages labeled by F4/80 staining in the colon’s lamina propria and reduced the expression of inflammatory mediators such as IL-6, TNF-α, IL-1β, monocyte chemoattractant protein-1 (MCP-1), and inducible nitric oxide synthase (iNOS). In addition, in an in vitro study using LPS-induced RAW264.7 cells, we have confirmed that SVHRSP reduced the release of inflammatory cytokines. Thus, these data demonstrated that SVHRSP inhibited the recruitment of macrophages and decreased the release of inflammatory factors. Although the anti-inflammatory effect of SVHRSP was demonstrated in RAW264.7 macrophages, it should be noted that this immortalized cell line may not fully replicate the complexity of primary macrophages in vivo; nevertheless, the consistency with our in vivo data supported the crucial role of macrophage modulation.

The vagus nerve is a critical component of the bidirectional communication neuron pathway in the gut–brain axis. The vagus nerve exerts an anti-inflammatory effect mediated by the cholinergic anti-inflammatory pathway (CAIP). During inflammatory responses, efferent vagal fibers release acetylcholine (ACh), which acts as an endogenous ligand binding to α7 nicotinic acetylcholine receptors (α7nAChRs) on macrophages, which triggers CAIP and inhibits the release of inflammatory mediators [41,42]. In this study, the concentration of ACh was decreased in DSS-exposed mice, indicating the inactivation of CAIP. However, the expression of α7nAChR was increased in DSS-exposed mice, and this may be due to the compensatory effects of chronic inflammation. SVHRSP administration led to elevated ACh levels and a concomitant reduction in α7nAChR expression. Consistent with these results, SVHRSP intervention decreased the expression of α7nAChR in LPS-stimulated RAW264.7 cells. These data suggested that SVHRSP exerted anti-colitis effects by acting on α7nAChR. To further confirm that α7nAChR is a target of SVHRSP, we established a chronic colitis model using α7nAChR KO mice. As predicted, the SVHRSP attenuated the protective effect in DSS-exposed α7nAChR KO mice.

Our results demonstrate that SVHRSP alleviated intestinal inflammation via an α7nAChR-dependent mechanism, as evidenced by its lost efficacy in α7nAChR-knockout mice and its direct binding potential revealed by molecular docking. The α7nAChR, as a homomeric ligand-gated ion channel, is broadly expressed in both the central nervous system (e.g., hippocampus and hypothalamus) and peripheral tissues such as the enteric nervous system and immune cells [43,44,45]. Its activation by endogenous acetylcholine or exogenous agonists modulates diverse physiological processes, including calcium influx, neurotransmitter release, synaptic plasticity, and anti-inflammatory responses [44].

The JAK2/STAT3 signaling pathway participates in the vagal anti-inflammatory pathway [27]. The anti-inflammatory effects of α7nAChR activation involve rapid JAK2 recruitment following acetylcholine binding. The receptor-coupled JAKs are activated by tyrosine phosphorylation close to each other, thereby attracting STAT3 molecules to aggregate and undergo tyrosine phosphorylation. The phosphorylated STAT3 forms a dimer and dissociates into the nucleus to regulate target genes [46,47]. In this study, SVHRSP promoted the phosphorylation of JAK2 and STAT3 in DSS-exposed WT mice. However, this effect was not observed in DSS-exposed α7nAChR knockout mice, indicating that the activation of JAK2/STAT3 by SVHRSP is α7nAChR-dependent.

Given the widespread expression of α7nAChR, the specific location where SVHRSP exerts its anti-inflammatory effect remains undetermined. Several non-mutually exclusive, peripheral mechanisms could plausibly explain our results. SVHRSP may act directly on α7nAChRs expressed on gut-resident macrophages, a scenario supported by our in vitro data in RAW264.7 cells and the molecular docking results indicating stable binding. This direct interaction could either activate the receptor or increase its sensitivity to endogenous acetylcholine (ACh). Alternatively, a plausible mechanism involves SVHRSP modulating the enteric nervous system (ENS)—the intrinsic nervous system of the gut. By acting on cholinergic neurons within the ENS, SVHRSP could enhance the local release of ACh, which in turn activates α7nAChR on nearby macrophages in a paracrine fashion to suppress inflammation. This model aligns with our observations of normalized ACh levels and α7nAChR expression following SVHRSP treatment. Additionally, other peripheral mechanisms may contribute to the therapeutic effects of SVHRSP, such as potential modulation of the gut microbiota [48]. While a central vagal pathway contribution cannot be entirely ruled out, the evidence suggests a predominantly peripheral site of action. Nevertheless, our data supported the α7nAChR-JAK2/STAT3 pathway as a primary mechanism, although we cannot fully rule out the contribution of other pathways.

Although the DSS-induced colitis model exhibits systemic immune activation and leukocytosis, the present study did not assess the potential effects of SVHRSP on peripheral leukocyte counts. However, potential concerns regarding systemic hematological toxicity of SVHRSP can be alleviated by our prior toxicological data (Supplementary Table S6, which demonstrated that subacute administration of SVHRSP in healthy rats did not induce any significant alterations in hematological parameters, including total and differential white blood cell counts.

Importantly, the well-established safety profile of SVHRSP, as confirmed in regulatory-approved toxicology studies, allows us to conclude that its anti-colitis efficacy at the therapeutic dose of 400 μg/kg/day is mediated by a specific activation of the α7nAChR–JAK2/STAT3 pathway, rather than by nonspecific immunosuppression or systemic toxicity. It should be noted that this study was conducted exclusively in male mice. This design choice was made to control for the potential confounding effects of the estrous cycle on inflammatory responses, thereby reducing initial experimental variance. Future investigations incorporating both female and male subjects will be essential to determine the broader applicability and potential sex-dependent efficacy of SVHRSP.

## 5. Conclusions

We provided compelling evidence that the novel synthetic neuroactive peptide SVHRSP alleviates DSS-induced chronic colitis via the α7nAChR-mediated JAK2/STAT3 signaling pathway. Our findings suggest that drugs possessing both anti-inflammatory and antioxidant properties—particularly those with neuroactive effects—may confer protection against intestinal inflammation. This work strengthens the rationale for exploring neuroimmune pathways in IBD and provides a foundational basis for repurposing neuroprotective peptides to treat inflammatory gut disorders.

## Data Availability

The original contributions presented in this study are included in the article/Supplementary Material. Further inquiries can be directed to the corresponding author.

## Supplementary Materials

The following supporting information can be downloaded at: https://www.mdpi.com/article/10.3390/antiox14111296/s1, Figure S1: Mouse tail identification of α7 nicotinic acetylcholine receptor (α7nAChR)-pure knockout mice; Table S1: Primers used for genotyping of α7 nicotinic acetylcholine receptor (α7nAChR)-pure knockout mice; Table S2: Disease activity index scoring; Table S3: Reaction mixture for RT-qPCR; Table S4: Temperature program used for RT-qPCR; Table S5: Primers sequence and annealing temperatures for RT-qPCR; Figure S2: Gating strategy and unstained control for macrophage analysis (corresponding to Main Figure 3C); Figure S3: Gating strategy and unstained control for macrophage analysis (corresponding to Main Figure 6H); Table S6: Complete blood count (CBC) analysis was performed after 14 days of subcutaneous administration of SVHRSP in male Sprague-Dawley rats (*n* = 5).

## Abbreviations

The following abbreviations are used in this manuscript:

IBD	inflammatory bowel diseases
SVHRSP	synthetic peptide-scorpion venom heat-resistant synthetic peptide
DSS	dextran sodium sulfate
α7nAChR	α7 nicotinic acetylcholine receptor
LPS	lipopolysaccharide
JAK2	Janus kinase 2
STAT3	signal transducer and activator of transcription 3
UC	ulcerative colitis
CD	Crohn’s disease
5-ASA	5-aminosalicylic acid
CAIP	cholinergic anti-inflammatory pathway
ACh	acetylcholine
IL-1	interleukin-1
TNF-α	tumor necrosis factor-alpha
HMGB1	high mobility group box 1
VNS	vagus nerve stimulation
HPA	hypothalamic–pituitary–adrenal
WT	wild-type
DAI	disease activity index
FITC	fluorescein isothiocyanate
ELISA	enzyme-linked immunosorbent assay
RT-qPCR	reverse transcription quantitative polymerase chain reaction
GAPDH	glyceraldehyde-3-phosphate dehydrogenase
ZO-1	zonula occludens-1
NOX2	nicotinamide adenine dinucleotide phosphate oxidase 2
HRP	horseradish peroxidase
MUC2	mucin 2
8-OHdG	8-hydroxy-2′-deoxyguanosine
DAB	3, 3′-diaminobenzidine
AOD	average optical density
PBS	phosphate-buffered Saline
FBS	fetal bovine serum
CAT	catalase
H_2_O_2_	hydrogen peroxide
SOD	superoxide dismutase
WST-1	water-soluble tetrazolium salt-1
O_2_•^−^	superoxide anion
MDA	malondialdehyde
TBA	thiobarbituric acid
MPO	myeloperoxidase
DHE	dihydroethidium
IL-6	interleukin-6
IL-1β	interleukin-1 beta
SEM	standard error of the mean
MCP-1	monocyte chemoattractant protein-1
iNOS	inducible nitric oxide synthase
ROS	reactive oxygen species
GI	gastrointestinal
SSRI	selective serotonin reuptake inhibitor
TBI	traumatic brain injury
