# Supplementary material for: Scorpion Venom Heat-Resistant Synthetic Peptide Alleviates DSS-Induced Colitis via α7nAChR-Mediated Modulation of the JAK2/STAT3 Pathway
Source: Antioxidants (Basel). 2025 Oct 28;14(11):1296. doi: 10.3390/antiox14111296 (PMC12649519; doi:10.3390/antiox14111296)
Supplement: Supplementary file 1 [file antioxidants-14-01296-s001.zip › antioxidants-3910637-supplementary.pdf]

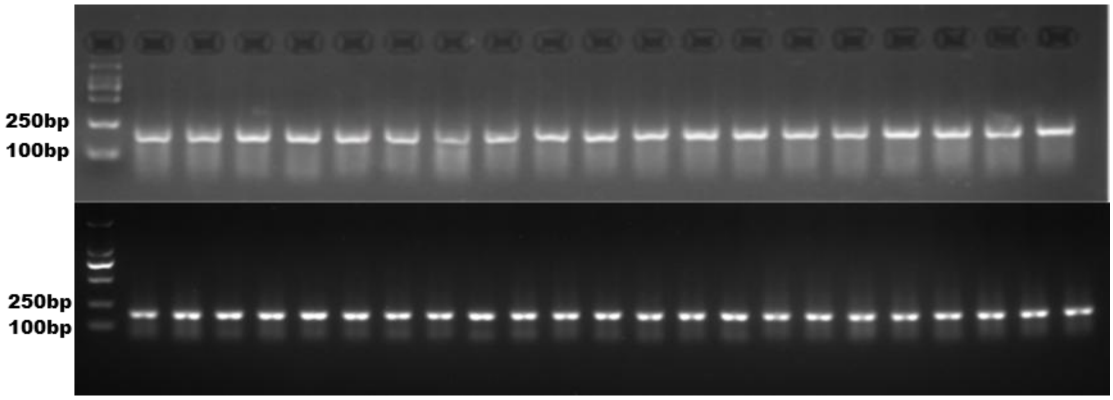

Figure S1. Mouse tail identification of α7 nicotinic acetylcholine receptor (α7nAChR)-pure knockout mice. Heterozygote = 187 bp and 390 bp, Wild type = 390 bp, Mutant = 187 bp.

Table S1 Primers used for genotyping of α7 nicotinic acetylcholine receptor (α7nAChR)-pure knockout mice.

**Primer name**	**Primer (5′-3′)**
Common	TTC CTG GTC CTG CTG TGT TA
Wild type Reverse	ATC AGA TGT TGC TGG CAT GA
Mutant Reverse	CCC TTT ATA GAT TCG CCC TTG

Table S2 Disease activity index scoring.

**Score**	**Weight loss (%)**	**Stool consistency**	**Blood stool**
0	No less	Normal	No blood
1	1-5	Fluffy and still formed	
2	5-10	Very soft	Blood visible in the stool
3	10-20	Loose and unmolded	
4	> 20	Watery diarrhea	Gross bleeding

Table S3 Reaction mixture for RT-qPCR.

**Component**	**Per reaction**
ChamQ SYBR Color qPCR Master Mix	5 μL
Forward primer (10 pmol/μL)*	0.2 μL
Reverse primer (10 pmol/μL)*	0.2 μL
Nuclease-free water	3.6 μL
cDNA template (8 ng/μL)	1 μL

The asterisk (*) indicates that the primers used in this reaction correspond to the specific primer sequences listed in Table S5.

Table S4 Temperature program used for RT-qPCR.

Operation	Temperature (℃)	Duration	Cycles
Initial activation	95	30 sec	1
Denaturation	95	10 sec	40
Annealing	*	30 sec	
Melting curve	95	1 min	1
	60	30 sec	
	60-95 （+0.2/sec）	1 min	

The asterisk (*) indicates that the annealing temperature corresponds to the Tm value listed for each primer pair in Table S5.

Table S5 Primers sequence and annealing temperatures for RT-qPCR.

Gene	Forward Primer (5′-3′)	Reverse Primer (5′-3′)	Annealing temperature Tm (℃)
IL-6	GAGGATACCACTCCCAACAGACC	AAGTGCATCATCGTTGTTCATACA	61
TNF-α	CTGTCTACTGAACTTCGGGGTGAT	GGTCTGGGCCATAGAACTGATG	60
IL-1β	GCTGAAAGCTCTCCACCTCA	AGGCCACAGGTATTTTGTCG	59
MCP-1	CGGAACCAAATGAGATCAGAA	TGTGGAAAAGGTAGTGGATGC	61
iNOS	GACATTACGACCCCTCCCAC	ACTCTGAGGGCTGACACAAG	60
Occludin	GAGCTTACAGGCAGAACTAGAC	CAGCCATGTACTCTTCACTCTC	62
ZO-1	CATCTCCAGTCCCTTACCTTTC	CCTCCAGGCTGACATTAGTTAC	60
GAPDH	TGTAGACCATGTAGTTGAGGTCA	AGGTCGGTGTGAACGGATTTG	60

Abbreviations: IL-6, interleukin-6; TNF-α, tumor necrosis factor-alpha; IL-1β, interleukin-1β; MCP-1, monocyte chemoattractant protein-1; iNOS, inducible nitric oxide synthase; ZO-1, zonula occludens-1; GAPDH, glyceraldehyde-3-phosphate dehydrogenase.

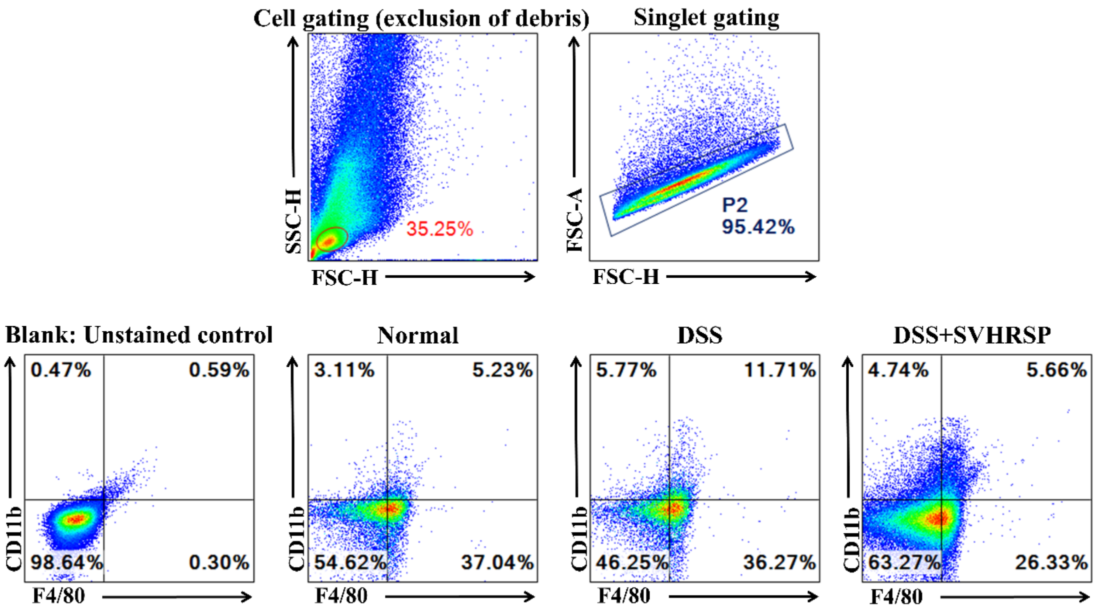

Figure S2. Gating strategy and unstained control for macrophage analysis (corresponding to Main Figure 3C). Cells were first gated on FSC-H vs SSC-H to exclude debris, followed by FSC-H vs FSC-A to select singlets. Representative dot plots show unstained control (Blank) and experimental groups (Normal, DSS, DSS+SVHRSP). Percentages indicate the proportion of $F4/80^{+}CD11b^{+}$ macrophages. **Abbreviations:** DSS, dextran sodium sulfate; SVHRSP, synthetic peptide-scorpion venom heat-resistant synthetic peptide; FSC-H, forward scatter-height; FSC-A, forward scatter-area; SSC-H, side scatter-height.

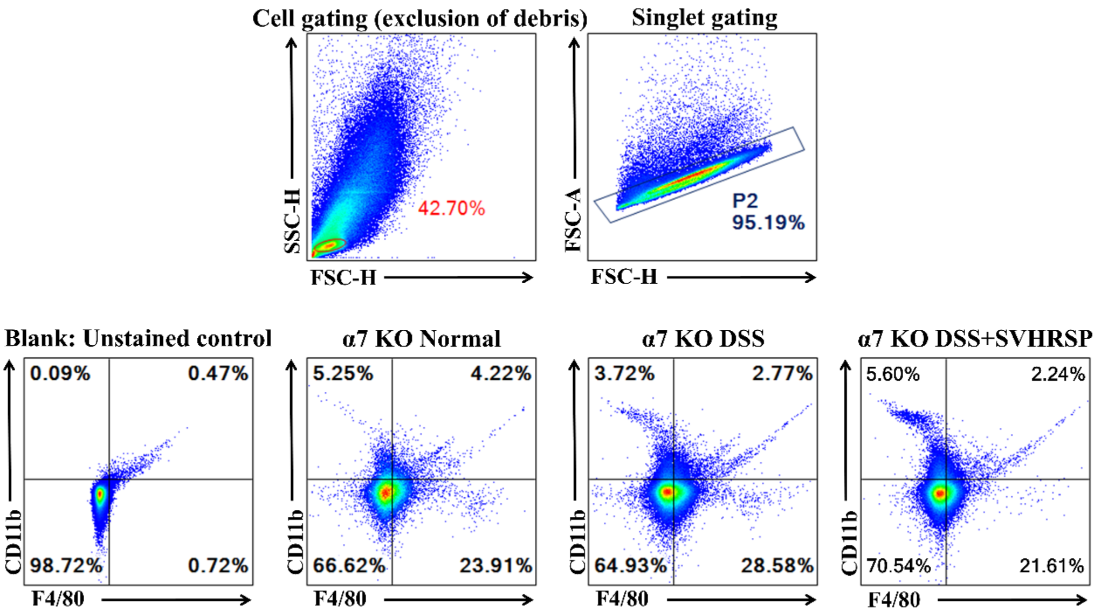

Figure S3. Gating strategy and unstained control for macrophage analysis (corresponding to Main Figure 6H). Cells were first gated on FSC-H vs SSC-H to exclude debris, followed by FSC-H vs FSC-A to select singlets. Representative dot plots show unstained control (Blank) and experimental groups (α7 KO Normal, α7 KO DSS, α7 KO DSS+SVHRSP). Percentages indicate the proportion of $F4/80^{+}CD11b^{+}$ macrophages. **Abbreviations:** α7 KO, α7 nicotinic acetylcholine receptor-pure knockout; DSS, dextran sodium sulfate; SVHRSP, synthetic peptide-scorpion venom heat-resistant synthetic peptide; FSC-H, forward scatter-height; FSC-A, forward scatter-area; SSC-H, side scatter-height.

Table S6 Complete blood count (CBC) analysis was performed after 14 days of subcutaneous administration of SVHRSP in male Sprague-Dawley rats (n = 5).

Parameter	0 mg/kg/day	20 mg/kg/day	60 mg/kg/day	200 mg/kg/day
Red Blood Cell Count ($\times 10^{12}$/L)	7.418 ± 0.17	7.310 ± 0.15	7.472 ± 0.11	7.520 ± 0.19
Hematocrit (%)	50.42 ± 0.99	48.86 ± 0.95	50.48 ± 0.68	51.08 ± 0.93
Hemoglobin (g/dL)	14.80 ± 0.28	14.20 ± 0.27	14.56 ± 0.19	14.62 ± 0.21
Mean Corpuscular Volume	68.02 ± 0.72	66.86 ± 0.74	67.58 ± 0.68	67.98 ± 0.72

(fL)				
Mean Corpuscular Hemoglobin (pg)	19.98 ± 0.19	19.48 ± 0.30	19.44 ± 0.22	19.44 ± 0.24
Mean Corpuscular Hemoglobin Concentration (g/dL)	29.34 ± 0.13	29.10 ± 0.34	28.80 ± 0.03	28.62 ± 0.13*
Reticulocyte Count (×10^9/L)	281.20 ± 23.61	248.04 ± 12.35	274.84 ± 10.83	285.84 ± 23.15
White Blood Cell Count (×10^9L)	8.018 ± 0.56	7.906 ± 0.67	8.342 ± 0.69	8.106 ± 0.42
Neutrophil Count (×10^9/L)	0.836 ± 0.14	0.808 ± 0.04	0.846 ± 0.12	1.004 ± 0.13
Lymphocyte Count (×10^9/L)	6.680 ± 0.47	6.740 ± 0.63	7.024 ± 0.61	6.584 ± 0.49
Monocyte Count (×10^9/L)	0.244 ± 0.03	0.152 ± 0.02	0.202 ± 0.04	0.252 ± 0.04
Basophil Count (×10^9/L)	0.010 ± 0.00	0.004 ± 0.00	0.008 ± 0.00	0.008 ± 0.00
Eosinophil Count (×10^9/L)	0.054 ± 0.01	0.070 ± 0.01	0.114 ± 0.02*	0.104 ± 0.02
Platelet Count (×10^9/L)	1091.2 ± 40.6	1182.6 ± 58.9	1150.6 ± 106.3	1161.4 ± 34.5
Mean Platelet Volume (fL)	12.04 ± 0.12	12.22 ± 0.21	12.14 ± 0.20	12.44 ± 0.21

Data were presented as mean ± standard error of the mean (SEM). *$p<0.05$ versus 0 mg/kg/day group.
